# Supplementary material for: Effectiveness and promising behavior change techniques of interventions targeting energy balance related behaviors in children from lower socioeconomic environments: A systematic review
Source: PLoS One. 2020 Sep 1;15(9):e0237969. doi: 10.1371/journal.pone.0237969 (PMC7462275; doi:10.1371/journal.pone.0237969)
Supplement: S1 Table — (DOCX) [file pone.0237969.s002.docx]

**S1 Table. Search strategy applied in Pubmed.**

| #1 | "sedentary behaviour" OR "sedentary behaviours" OR "sedentary behavior" OR "sedentary behaviors" OR "sedentary time" OR "sedentary lifestyle" OR "sitting time" OR "prolonged sitting" OR "computer time" OR "computer use" OR "screen time" OR "screen-time" OR "sedentary activity" OR "sedentary activities" OR television OR gaming OR "cell phone" OR “mobile phone” |
| --- | --- |
| #2 | "physical activity” OR “physical activities” OR exercis* OR sport OR sports OR active OR activities OR activity OR walking OR cycling OR running OR recreation OR play |
| #3 | nutrition OR food OR diet OR intake OR beverages OR “sugar sweetened beverages” OR “energy drinks” OR “energy drink” OR “carbonated beverage*” OR “carbonated drink*” OR “fruit vegetable juice” OR “fast foods” OR “fast food” OR candy OR snack OR snacking OR snacks OR “snack food” OR "soft drink" OR fruit OR fruits OR vegetables OR vegetable OR drinks OR soda OR drinking OR breakfast OR supper OR lunch OR meal OR dinner OR tea OR fat OR “5-a-day” OR eating |
| #4 | #1 OR #2 OR #3 |
| #5 | Health promotion[MeSH:noexp] |
| #6 | "health promotion"[MeSH] OR "health behaviour" OR "health behavior" OR ("policy" and ("social" OR "school" OR "food" OR public OR urban OR environmental OR fiscal)) OR "urban planning" OR "city planning" OR "built environment" OR "social environment" OR "physical environment" OR "cultural environment" OR "urban environment" OR "school environment" OR neighbourhood OR neighborhood OR community OR societal OR "social interventions" OR "community interventions" OR "obesogenic environment" OR "individual level" OR "lifestyle" OR "individual" OR tax OR taxes OR subsid* OR price* OR "health education" OR "social marketing" OR (diet AND (advice OR counseling)) OR (exercise AND (advice OR counseling)) OR "weight management" OR "cash transfer*" OR "lifestyle counselling" OR "behavioural counseling" OR "behavioral counseling" OR "exercise on prescription" OR exercise OR "health trainer*" OR school OR workplace OR campaign* OR "access to facilities" OR "green space" OR "walking ability" OR "food label*" OR "food advert*" |
| #7 | #5 OR #6 |
| #8 | evaluat* OR effective* OR intervention OR "RCT" OR experiment* OR placebo OR random* OR trial OR "quasi-experiment*" OR "pre-test" OR "post-test" OR trial OR "time series" OR evaluat* OR intervention* OR "before and after" OR "community trial" OR "non-randomized" OR "non-randomised" OR repeat* OR "clinical trial" OR "latin square" OR "pseudo-experimental" OR "pseudo-randomized" OR "pseudo-randomised" OR "quasi-randomized" OR "quasi-randomised" OR "quasiexperiment*" OR pretest OR posttest OR nonrandomized OR nonrandomised OR pseudoexperimental OR pseudorandomized OR pseudorandomised OR quasirandomized OR quasirandomised |
| #9 | "clinical trial" OR "clinical trials as topic" OR "randomized controlled trial" OR "randomized controlled trials as topic" OR "random allocation" OR "double-blind method" OR "single-blind method" OR "cross-over studies" OR "evaluation studies" OR "evaluation studies as topic" OR controlled trial OR “control group” |
| #10 | (“population level” OR “population based” OR “population orientated” OR “population oriented” OR “community level” OR “community based” OR “community orientated” OR “community oriented” OR school OR “school-based” OR institution OR institutional OR class) AND (intervention* OR prevention OR policy OR policies OR program* OR project* OR program OR programs OR programme OR programmes OR change OR changing OR trial OR trials OR approach OR approaches) |
| #11 | #8 OR #9 OR #10 |
| #12 | #4 AND #7 AND #11 |
| #13 | "socioeconomic factors" OR income OR "socioeconomic" OR "socio-economic" OR "social economic" OR "SES" OR "low-income population" OR "Household income” OR “disadvantage*” |
| #14 | #12 AND #13 |
| #15 | #12 AND #13 Filters: Publication date from 2000/01/01 to 2019/08/31; Child: birth-18 years |
