# Supplementary material for: Effectiveness and promising behavior change techniques of interventions targeting energy balance related behaviors in children from lower socioeconomic environments: A systematic review
Source: PLoS One. 2020 Sep 1;15(9):e0237969. doi: 10.1371/journal.pone.0237969 (PMC7462275; doi:10.1371/journal.pone.0237969)
Supplement: S2 Table — (DOCX) [file pone.0237969.s003.docx]

**S2 Table. Quality assessment tool.**

| **Article number:** |  |  | **Primary author:** |  | **Year of publication** |  |  |  |  |
| --- | --- | --- | --- | --- | --- | --- | --- | --- | --- |

**A. SELECTION BIAS**

| Q1 Representative^1^ | Very likely | Somewhat likely | Not likely |  |  |
| --- | --- | --- | --- | --- | --- |
| Q2 Participation^2^ | 80-100% | 60-79% | Less than 60% / can’t tell | |  |
| RATING | STRONG | MODERATE | WEAK |  |  |

^1^ Related to participating schools. Do the selected schools represent the target population.
^2^ Related to participating children.

**B. STUDY DESIGN**

| Design | RCT | Controlled clinical trial |
| --- | --- | --- |
| Randomized | YES | NO |
| Described as randomized | YES | NO |
| Method of randomization | YES | NO |
| Appropriate method | YES | NO |
| RATING | STRONG |  |

**C. CONFOUNDERS**^3^

| Q1 Group differences | NO | YES | Can’t tell |
| --- | --- | --- | --- |
| Q2 Controlled | Baseline outcome, sex, age, ethnicity, SES | Baseline outcome | No relevant confounders / can’t tell |
| RATING | STRONG | MODERATE | WEAK |

^3^ If Q1 is NO, no correction at Q2 could still mean STRONG. If Q1 is YES and no correction: WEAK. If Q1 is YES **and** correction: MODERATE

**D. BLINDING^4^**

| Q1 Assessors^5^ | YES |  | NO / can’t tell |
| --- | --- | --- | --- |
| Q2 Participants | YES |  | NO / can’t tell |
| RATING | STRONG | Combi YES/NO  MODERATE | WEAK |

^4^ Note if multiple measurement tools have different quality
^5^ If it is a self-reported questionnaire, the child is participant and assessor

**E. DATA COLLECTION METHODS**

| Q1 Valid | Objective or validity >0.7 | YES – NO | NO / can’t tell |
| --- | --- | --- | --- |
| Q2 Reliable | Objective or reliability >0.7 | NO – YES | NO / can’t tell |
| RATING | STRONG | MODERATE | WEAK |

**F. WITHDRAWALS AND DROP-OUTS**

| Q1 Reported^6^ | YES/NA |  | NO / can’t tell |
| --- | --- | --- | --- |
| Q2 Completion | 80-100% | 60-79% | <60% / can’t tell |
| RATING | STRONG | MODERATE | WEAK |

^6^ Concerns reported **reasons** for drop-out

**G. INTERVENTION INTEGRITY**

| Q1 Intended intervention | 80-100% | 60-79% | <60% / can’t tell |
| --- | --- | --- | --- |
| Q2 Consistency measured | YES | NO | Can’t tell |
| RATING | STRONG | MODERATE | WEAK |

**H. ANALYSES**

| Q1 Allocation / analysis unit^7^ | Similar |  | Not similar / can’t tell |
| --- | --- | --- | --- |
| Q2 Intention to treat | YES |  | NO / can’t tell |
| Q3 Subjects = 10x variables | YES |  | NO / can’t tell |
| Q4 Point estimate^8^ | YES |  | NO / can’t tell |
| RATING | STRONG | Combi^9^  MODERATE | WEAK |

^7^Level of allocation and analysis should be the same for a strong analysis (multi-level), otherwise weak

^8^ i.e. beta [95% CI] but not t-test

^9^e.g. Q1 not similar, but Q2, Q3 and Q4 ‘yes’

**GLOBAL RATING**

| RATING | STRONG | MODERATE | WEAK |  |  |
| --- | --- | --- | --- | --- | --- |

**STRONG:** **MAX ONE** WEAK SCORE AND **MAXIMUM 2 MODERATE** SCORES

**MODERATE:** MAX. **TWO** WEAK SCORE

**WEAK:** **>2 WEAK** SCORE
