# Supplementary material for: Effectiveness and promising behavior change techniques of interventions targeting energy balance related behaviors in children from lower socioeconomic environments: A systematic review
Source: PLoS One. 2020 Sep 1;15(9):e0237969. doi: 10.1371/journal.pone.0237969 (PMC7462275; doi:10.1371/journal.pone.0237969)
Supplement: S3 Table — Bold are studies that are effective in changing physical activity, dietary behavior and/or sedentary behavior. BM = behavioral modification, FMS = fundamental movement skills, + = strong score, +/- = moderate score - = weak score. (DOCX) [file pone.0237969.s004.docx]

**S3 Table. Quality assessment scores of included studies and its items.**

| **Quality items →**  **Author, Year ↓** | **Analyses** | **Intervention integrity** | **Withdrawals and drop-outs** | **Data collection methods** | **Blinding** | **Confounders** | **Study design** | **Selection bias** | **Overall quality score** |
| --- | --- | --- | --- | --- | --- | --- | --- | --- | --- |
| Alaimo et al., 2015 [43] | + | - | - | +/- | - | +/- | + | - | - |
| Ashfield-Watt et al., 2008 [51] | +/- | + | - | + | - | +/- | + | - | - |
| **Bastian et al., 2015 [52]** | + | - | - | + | - | +/- | + | +/- | - |
| Beyler et al., 2014 [44] | + | - | +/- | + | - | - | + | +/- | - |
| Bohnert and Ward, 2013 [42] | - | +/- | - | - | - | - | + | - | - |
| **Breslin et al., 2012 [54]** | - | - | - | - | - | - | + | - | - |
| Colín-Ramírez et al., 2010 [55] | - | - | + | +/- | - | +/- | + | - | - |
| Dunton et al., 2015 [41] | +/- | - | - | - | - | - | + | - | - |
| Gatto et al., 2017 [49] | +/- | - | + | +/- | - | - | + | +/- | - |
| Gittelsohn et al., 2010 [40] | +/- | - | +/- | - | - | - | + | +/- | - |
| **Harrison et al., 2006 [56]** | + | + | + | +/- | - | + | + | +/- | + |
| Keihner et al., 2017 [35] | - | - | + | - | - | +/- | + | - | - |
| Lent et al., 2014 [45] | + | - | - | + | - | - | + | - | - |
| Madsen et al., 2013 [39] | + | - | + | + | - | +/- | + | +/- | +/- |
| **Mendoza et al., 2017 [36]** | + | - | + | + | - | - | + | - | - |
| Neumark-Sztainer et al., 2009 [46] | - | - | + | - | - | + | + | - | - |
| Nollen et al., 2014 [34] | - | - | + | - | - | - | + | - | - |
| **Salmon et al., 2008 [FMS]**^2^ [57] | + | - | + | +/- | - | - | + | +/- | + |
| Salmon et al., 2008 [BM]^2^ [57] | + | - | + | +/- | - | - | + | +/- | + |
| Salmon et al., 2011 [58] | +/- | +/- | + | +/- | - | - | + | +/- | +/- |
| Slusser et al., 2010 [38] | +/- | - | + | +/- | - | - | + | - | - |
| Springer et al., 2012 [47] | +/- | - | + | +/- | - | +/- | + | - | - |
| Trude et al., 2018 [5] | + | +/- | +/- | - | - | - | + | - | - |
| **Van de Gaar et al., 2014 [59]** | +/- | - | + | +/- | - | +/- | + | +/- | +/- |
| **Vander Ploeg et al., 2014 [53]** | + | - | - | +/- | - | - | + | +/- | - |
| Wang et al., 2019 [37] | + | +/- | + | +/- | - | - | + | - | - |
| Wells et al., 2014 [48] | + | - | - | + | - | - | + | +/- | - |

**Bold** are studies that are effective in changing physical activity, dietary behavior and/or sedentary behavior. BM=behavioral modification, FMS=fundamental movement skills, +=strong score, +/-=moderate score -=weak score.
