# Supplementary material for: Effectiveness and promising behavior change techniques of interventions targeting energy balance related behaviors in children from lower socioeconomic environments: A systematic review
Source: PLoS One. 2020 Sep 1;15(9):e0237969. doi: 10.1371/journal.pone.0237969 (PMC7462275; doi:10.1371/journal.pone.0237969)
Supplement: S4 Table — ↔ indicates intervention overall not effective on at least 75% of the outcomes within the behavior, ↑ indicates intervention overall effective on at least 75% of the outcomes within the behavior,? = unclear on the amount of outcome measures in the questionnaire. BM = behavioral modification, CG = control group, DB = dietary behavior, FMS = fundamental movement skills, FV = fruits and vegetables, IG = intervention group, MPA = moderate physical activity, MVPA = moderate to vigorous physical activity, PA = physical activity, SB = sedentary behavior, ST = screen time, T0 = baseline, VPA = vigorous physical activity, vs = versus. *ST is reported when a study specifically focused on ST instead of on SB in general. (DOCX) [file pone.0237969.s005.docx]

**S4 Table. Behavior change techniques and outcomes of the included articles, sorted by methodological quality.**

| Study and methodological quality | Summary intervention and applied behavior change techniques (BCTs) | Measurement tools | Summary of results |
| --- | --- | --- | --- |
| Harrison et al., 2006[56]; strong quality rating | 10 lessons aimed to increase physical activity at the expense of screen time, based on concepts for behavioral change (self-control (of screen time), self-monitoring, budgeting and goal setting).  **1.1 Goal setting (behavior)** – self-monitoring, budgeting and goal setting practiced to decrease screen time and increase physical activity  **1.2 Problem solving** – self-control of physical activity was enhanced by addressing barriers to increased activity and ways of overcoming these barriers  **1.4 Action planning** – children were encouraged to set personal targets  **2.1 Monitoring of behavior by others without feedback** – parents verify behavior by signing diaries  **2.3 Self-monitoring of behavior** – pupil diaries to record leisure time activity/screen time  **3.1 Social support (unspecified)** – parents were encouraged in writing to support children in their attempts to switch off and get active  **4.1 Instruction on how to perform a behavior** – this is the aim of the ten lessons  **6.2 Social comparison** – the diaries were also used for minor intra- and inter-school competitions  **8.2 Behavior substitution** – children were required to reflect on how they spend their leisure time and challenged to identify realistic alternatives to television viewing and computer game usage  **10.2 Material reward (behavior)** – one point was awarded for every 5 min of physical activity  **10.3 Non-specific reward** – small prizes were used to reinforce individual improvement and class achievement  **14.1 Behavior cost** – one point deducted for every 15 min of screen time  **Community participation/involvement –** parents were encouraged in writing to support children and verify behavior  **Knowledge transfer** – aim of the 10 lessons | **PA**: 1-day Previous Day Physical Activity Recall (PDPAR). Administered on one Sunday and two other weekdays: number of 30 minute blocks/day of MVPA (breathlessness and sweating).  **ST***: 1-day Previous Day Physical Activity Recall (PDPAR). Administered on one Sunday and two other weekdays: number of 30 minute blocks/day of screen time per day (TV/video/dvd, computer game usage). | **PA**: ↑  At T1 (post-intervention) compared to T0, significant increase in MVPA in the intervention group vs the control group (adjusted difference β=0.84 (95%CI: 0.11, 1.57)).  **ST**: ↔  No significant effects |
| Madsen et al., 2013[39]; moderate quality rating | After-school soccer program, also including creative writing and service learning. Primary goal of building competencies and skills that support students' overall development. Includes the train-the-trainer model.  **4.1 Instruction on how to perform the behavior**, and  **6.1 Demonstration of the behavior**, and  **8.1 Behavioral practice/rehearsal** – students spend 2-3 days per week in soccer drills or games for up to 2 hours each day | **PA**: Accelerometer: after school and Saturday MVPA minutes | **PA**: ↔  No significant effects |
| Salmon et al., 2011[58]; moderate quality rating | Based on Switch-play. Six lessons of the BM-arm used and adapted; targeting screen behaviors and physical activity directly as well as through mediators of change.  **Knowledge transfer** – education and awareness training on physical activity and health – the beginning of each subsequent lesson involved a summary and brief discussion of what was learnt in the previous lesson | **PA**: Self-reported survey: physical activities (min/week; 30 different activities) on weekdays and weekend days, to calculate min/day in MVPA  **ST**: Self-reported survey: screen behaviors (minutes/hours; TV viewing, playing electronic games, computer use) on weekdays and weekend days. | **PA**: ↔  No significant effects, also when stratified for gender  **ST**: ↔  No overall effects for screen behaviors. At T1 (post-intervention) compared to T0 moderation effects of gender: favorable intervention effects on weekend screen time in boys β=-0.62 (95%CI:  -1.15, -0.1) but not in girls β=0.13 (95%CI: 0.01, 0.25). |
| van de Gaar et al., 2014[59]; moderate quality rating | Implementation of a 'water campaign' at schools, using social marketing, lessons at school, integrated community activities to promote water consumption.  **3.2 Social support (practical)** – parents responsible for giving the child his/her water bottle to school  **5.1 Information about health consequences** – promotional material: ‘water is the best thing I can give to my child!’  **7.1 Prompts/cues** – pimp up your water bottle – pimp up your water jug  **8.1 Behavioral practice/rehearsal**, and  **8.3 Habit formation** – taking a water break during physical education lessons  **9.1 Credible source** – water show provided by children’s role models – promotion by water ambassadors  **12.5 Adding objects to the environment** – provision of free water bottles – provision of free water at school  **Community participation/involvement** – parents are responsible for giving the child his/her water bottle to school – water magazine for mothers – promotional material: ‘water is the best thing I can give to my child!’  **Knowledge transfer** – storytelling about promoting water consumption – educational water lessons – water magazine for mothers  **Active learning** – fun games involving water consumption | **DB**: Child- and parent self-report: children's sugar sweetened beverages (SSB) intake (%every day, liters and number of servings). Observations: %SSB brought to school | **DB**: ↑  At T1 (post-intervention compared to T0, parent report: average SSB intake (liters) β=-0.19 (95%CI=-0.28, -0.10); SSB servings β=-0.54 (95%CI=-0.82, -0.26). No significant effects for child report. Observation report: %SSB brought to school β=0.51 (95%CI=0.36, 0.72). |
| Alaimo et al. 2015[43]; weak quality rating | A multi-component initiative including school, community and social marketing elements, designed to improve dietary behaviors and increase physical activity among children. Project FIT has a school- and a social marketing component.  **4.1 Instruction on how to perform a behavior**, and  **6.1 Demonstration of the behavior**, and  **8.1 Behavioral practice/rehearsal** – healthy eating coaching; taste testing of healthy foods  **7.4 Remove access to the reward** – help teachers move away from using food as rewards  **12.1 Restructuring the physical environment** – improvements of school policies, programs and environments  **12.5 Adding objects to the environment** – non-food reward boxes  **Knowledge transfer** – nutrition education – health messages | **DB**: Questionnaire with questions adapted from the School Physical Activity and Nutrition Questionnaire and the 4th grade Nutrition Education Survey; Frequency of consumption of common foods (whole grain cereals, vegetables, beans, fruits, fruit juice); Plate consumption for school lunches (or plate waste) | **DB**: ↔  At T1 (after first year): No significant effects.  At T2 (after second year): whole grain breads frequency increased (times/d) (β=0.76; 0.06, 1.46) in intervention group vs^#^ the control group; frequency of vegetables intake increased (β=0.90; 0.11, 1.69),  Fruit intake increased (times/d) (β=1.02; 0.33, 1.7);  Fruit servings increased (β=2.16; 1.01, 4.62). |
| Ashfield-Watt et al. 2008[51]; weak quality rating | Provision of free seasonal fruits to schools.  **12.5 Adding objects to the environment** – seasonal fruits were provided  **Community participation/involvement** – distribution of the fruits within schools was undertaken by the children | **DB**: Day in the Life Questionnaire; (diet recall methodology): fruit intake (at school and total fruit intake) | **DB**: ↔  At T1 (post-intervention) compared to T0*: significantly higher fruit intake in the intervention group vs the control group (0.39 more pieces/school day). At T2 (6 week follow-up) compared to T1: significantly lower total fruit intake in the intervention group vs the control group. |
| Bastian et al. 2015[52]; weak quality rating | APPLE schools is a school-based health promotion intervention working with the Comprehensive School Health (CSH) approach. A school health facilitator is placed at the school to work with students, teachers, parents and the community to implement health promotion strategies that meet the school’s needs. Examples are the organization of extra activities during and after school hours and improved access to after school PA facilities and programs; school-wide activities are organized for students and parents; traffic safety is increased to support/promote active transport; monthly newsletters describing affordably, easily accessible and seasonally appropriate activities for children outside of school.  **4.1 Instruction on how to perform the behavior**, and  **6.1 Demonstration of the behavior**, and  **8.1 Behavioral practice/rehearsal** – a variety of enjoyable activity choices were offered weekly  **12.1 Restructuring the physical environment** – increasing traffic safety to promote and support active transportation – improve access to after-school physical activity facilities and programs  **Community participation/involvement** – student/parent activities – monthly school newsletters  **Knowledge transfer** – monthly school newsletters describing activities for children | **PA**: Daily physical activity using a pedometer | **PA**: ↑  At T1 (post-intervention) compared to T0 daily step counts increased 10.5% more on school days (β=1221 steps/day; 95%CI: 306, 2135) and 21.6% more on weekend days (β=2001 steps/day; 95%CI: 600, 3402) in the intervention group vs the control group. Hourly step counts increased 15.8% more during non-school hours (β=137 steps/hour; 95%CI: 31, 242) in the intervention group vs the control group. |
| Beyler et al., 2014[44]; weak quality rating | Playworks places full-time coaches at low-income schools to engage more children in physical activities and related life skills. The coaches are trained and supervised by Playworks staff. The coaches organize games and activities during recess, work with teachers to engage students in physical activity during class game time, and coordinate a junior coach program where older students help monitor recess periods.  **1.2 Problem solving –** conflict resolution  **4.1 Instruction on how to perform a behavior**, and  **6.1 Demonstration of the behavior**, and  **8.1 Behavioral practice/rehearsal** – full-time coaches organize games and activities during recess  **Community participation/involvement** – youth adults as coaches | **PA:** Accelerometer (subgroup): Intensity counts during recess; steps during recess; time in MVPA; time in MPA. Survey for teachers and students: percentage of students participating in activities making them sweat and breath hard, percentage of teachers agreeing their students participated in activities making them sweat and breath hard. | **PA**: ↔  At T1 (post-intervention) compared to T0 (baseline) teacher-reported levels of student physical activity during recess increased in the intervention group vs the control group (effect size: 32.4±0.85). No significant effects for accelerometer data and student survey. |
| Bohnert and Ward, 2013[42]; weak quality rating | GIG is a 30-week program with 10x3 modules, in which each module covers a different sport, health and leadership topic. The sessions are led by a GIG coach and involve physical instruction and physical activities, and education (health, nutrition, leadership, life skills)  **4.1 Instruction on how to perform a behavior**, and  **6.1 Demonstration of the behavior**, and  **8.1 Behavioral practice/rehearsal** – physical instruction and energetic activity through traditional and non-traditional sports and fitness activities  **10.2 Material reward (behavior)** – small prize for the girl who worked hardest at each session  **10.4 Social reward** – girl of the day award for the girl who worked the hardest at each session  **12.5 Adding objects to the environment** – a healthy snack or meal is provided at every session  **15.1 Verbal persuasion about capability** – empowering the girls to believe that they can make healthy choices as well as promoting self-control around health and life choices  **Community participation/involvement** – take-home materials for families to reinforce program messages  **Knowledge transfer** – health education, nutrition education, and leadership and life skills topics | **PA**: Questionnaire on PA behavior (frequency of sport participation of at least 20 min high intensity; frequency of strengthening exercises)  **DB**: Questionnaire on nutrition behavior (vegetable intake; intake of snacks) | **PA**: ↔  No significant effects  **DB**: ↔  No significant effects |
| Breslin et al., 2012[54]; weak quality rating | Sport for LIFE is a 12-week program aimed to increase knowledge and understanding about the benefits and importance of physical activity and healthy nutrition. Each week for 1 hour, children were taught activities on the effects of physical activity on health, fun games for indoors and outdoors and nutrition. The program ended with a physical activity festival.  **4.1 Instruction on how to perform a behavior**, and  **6.1 Demonstration of the behavior**, and  **8.1 Behavioral practice/rehearsal** – children were taught activities on the effects of physical activity on health, fun games for indoors and outdoors  **5.1 Information about health consequences** – understanding the benefits of regular participation in physical activity and sport and the importance of eating a healthy balanced diet  **9.1 Credible source** – the program ended with a physical activity festival with an invited Olympic gold medal winning guest in attendance | **PA**: Questionnaire with questions from the Physical Activity in Scotland Schoolchildren questionnaire (PASS): meeting 60-min MVPA recommendation. Accelerometer (subgroup): time spent in light, moderate and vigorous activities.  **DB**: Modified Child Nutrition Questionnaire. Items on:  food intake (non-core foods, fruit, vegetables, water, sweetened beverages without diet drinks), healthy eating behavior, environment (home or school environment fruit and vegetable consumption)  **SB**: Accelerometer (subgroup): time spent sedentary. Question from the Health Promotion Agencies National Children’s Survey: screen time (watching TV, watching DVD, playing video games) | **PA**: ↑  At T1 (post-intervention) compared to T0 significant main effects on levels of light (F(1,49)=23.650), moderate (F(1,49)=35.578), and vigorous activities (F(1,49)=24.337) in the intervention group vs the control group. **DB**: ↔  Significant mean reduction in non-core food from baseline to follow-up in the intervention group vs the control group (F(1,414)=6.969).  **SB**: ↑ **ST**: ↔  Significant main effects from baseline to follow-up on levels of sedentary activities (F(1,49)=5.585) in the intervention group vs the control group. No significant effects on screen time. |
| Colín-Ramírez et al., 2010[55]; weak quality rating | Intervention to increase PA, implementation at 3 levels. Individual level: classroom lessons and activity breaks. School level: increase duration of MVPA time in PE classes. Family level: involving family members in support and reinforcement of classroom lessons and recommendations for achieving a more active lifestyle for parents  **3.1 Social support (unspecified)** – each child received a book of activities to take home with exercises that corresponded to the lessons at school and was implemented with collaboration with his parents  **4.1 Instruction on how to perform a behavior**, and  **6.1 Demonstration of the behavior**, and  **8.1 Behavioral practice/rehearsal** – physical education classes  **8.1 Behavioral practice/rehearsal** – exercise breaks  **8.2 Behavior substitution** – a substitution of regular exercise during physical education classes for new ones that required moderate to vigorous energy output  **8.6 Generalization of target behavior** – take home book  **9.1 Credible source** – the lessons were given by health team  **Community participation/involvement** – parents received recommendations for achieving a more active lifestyle  **Knowledge transfer** – 30 min classroom lessons | **PA**: sPAN questionnaire. Items: how many days in the last week did you participate in MVPA and in MPA  **ST**: sPAN questionnaire. Items: hours of watching TV or video movies yesterday; usual amount of hours spent on computer use (away from school); usual amount of hours spending on playing video games | **PA**: ↔  No significant effects **ST**: ↔  No significant effects |
| Dunton et al., 2015[41]; weak quality rating | Parents could receive reimbursement up to $200 following enrolment of their school-aged child in a qualified after-school physical activity program. Program requirements include: aiming to encourage children to be active for 30 minutes of MVPA during a session; supervised by an adult, suitable for children, duration of at least 8 weeks with a minimum of one session per week.  **10.1 Material incentive**  **10.2 Material reward** – parents receive $200 if they sign their child up to a qualified physical activity program | **PA**: Assessed by self-report of parents (questionnaire; time allocation diary): enrolment rate in PA after-school programs; weekly participation in after-school programs, duration of enrolment (weeks) in after school programs, long-term enrolment pattern in after-school PA programs. Accelerometer: time spent in MVPA | **PA**: ↔  At T1 (post-intervention) compared to T0, the intervention group was more likely to be enrolled in an after-school program vs the control group OR=1.8 (95%CI: 0.2, 17.2). At T2 (6 week follow-up) compared to T0, the intervention group was less likely to continue enrolment at 6 week follow-up OR=0.8 (95%CI= 0.6,9.7). No significant effects in time spent in after school programs, or time spent in MVPA. |
| Gatto et al. 2017[49]; weak quality rating | LA Sprouts is a 12 week after-school gardening, nutrition/cooking intervention. Schools receive a raised bed planter box and an outdoor modular kitchen. Children receive nutrition lessons focused on increasing fruit and vegetable intake, and gardening lessons where children learn and participate in gardening activities. Children for example learn planting, harvesting, recycling and cooking.  **4.1 Instruction on how to perform a behavior**, and  **6.1 Demonstration of the behavior**, and **8.1 Behavioral practice/rehearsal** – interactive cooking/nutrition lessons – gardening lesson  **6.1 Demonstration of the behavior** – the snack was eaten in a family-style manner  **12.5 Adding objects to the environment** – raised bed planter boxes were places, gardening tools were provided, outdoor modular kitchen  **Community participation/involvement –** parallel classes were offered to parents bimonthly on mornings, evenings and weekends – monthly visits to a local farmers market  **Knowledge transfer** – children learned and participated in planting, growing, maintaining, and harvesting organic fruits and vegetables – gardening lessons also included identification of plants, square for gardening, seasonal crops, transplanting, recycling, composting, irrigation, and mulching. | **DB**: Dietary intake using the Block Kids Food Screener (“last week” version); 41 items: Nutrients: energy, protein, fat, carbohydrate, added sugar, dietary fibers. + Food/food groups: meat, diary, whole grains, vegetables, fruit/fruit juices, apples/bananas/oranges, lettuce/salads, green beans/peas, tomatoes. | **DB**: ↔  At T1 (post-intervention) compared to T0 intake of dietary fiber in intervention group increased by 0.4 grams/day (+3.4%), while this decreases in the control group by 2.0 grams/day (-16.5%). Both the intervention (-0.03 cup equivalent/day) and control group (-0.2 cup equivalent/day) had decreased vegetable intake from T0 to T1, but this was smaller in the intervention group (-3.7%), than the control group (-26.1%). |
| Gittelsohn et al., 2010[40]; weak quality rating | HFH aimed to increase the availability of healthy foods in stores, and promote healthier food choices and food preparation methods. Posters, educational displays and shelf labels were used in stores. Cooking demonstrations/taste tests were held four to six times per phase (4 phases in total; lasting 6-8 weeks each). Involvement of local producers and distributors for the provision of products and promotional items (cooking and taste tests).  **4.1 Instruction on how to perform a behavior**, and  **6.1 Demonstration of the behavior**, and  **8.1 Behavioral practice/rehearsal** – cooking demonstrations and taste tests were held with brochures and recipe cards distributed during the demonstrations/taste tests  **7.1 Prompts/cues** – local producers and their products were highlighted through the use of a ‘producer biography’, which was hung above their product in the produce section  **10.2 Material reward (behavior)** – gift certificates/giveaways  **12.1 Restructuring the physical environment** – changes in product distribution during specific phases  **12.5 Adding objects to the environment** – local producers and their products were highlighted through the use of a “producer biography”, which was hung above their product in the produce section  **Community participation/involvement** – phases were developed in a participative process with community members – decisions about which foods to focus on were (also) based on community workshops – two local producers and four local distributors were involved in the project **Knowledge transfer** – posters, educational displays, and shelf labels were used as educational tools | **DB**: Customer Impact Questionnaire (CIQ). Items: household meal patterns, child feeding, consume and prepare foods promoted by the intervention. Child Customer Impact Questionnaire (CCIQ). Items: food frequency.  24-hour dietary recall for both child and parent using the US Department of Agriculture’s five-step multiple pass methodology | **DB**: ↔  At T1 (post-intervention) compared to T0, significantly higher Healthy Eating Index (HEI) scores in the intervention group vs the control group (adjusted β=8.53; SE=3.49); increased grain HEI (adjusted β=1.83; SE=0.73); and total water consumption (adjusted β=2.72; SE=0.74). No significant effects on frequency of healthy or unhealthy food consumption. |
| Keihner et al. 2017[35]; weak quality rating | Power Play! is an education intervention during/after school and social marketing in the classroom cafeteria, after-school program, home, and community setting. Throughout 10 weeks, activities were conducted during/after school, including weekly FV/PA lessons and PA breaks; biweekly classroom promotions/taste tests; posters displayed in/around schools; and weekly nutrition materials for parents.  **1.1 Goal setting (behavior)** – set goals for increasing FV intake and levels of PA  **1.2 Problem solving** – identify barriers to eating FVs and PA, identify ways to overcome perceived barriers and build skills to reduce or eliminate barriers  **1.4 Action planning** – students make a plan to include at least five servings of FVs in their day  **1.5** **Review behavior goal(s)** – set goals for improving FV intake and levels of PA and assess their progress  **2.3 Self-monitoring of behavior** – students examine their current habits (FV, snack, breakfast)  **5.1 Information about health consequences** – identify benefits of eating FVs and being physically active  **6.2 Social comparison** – students identify their nutritional and PA habits and those of their classmates  **7.1 Prompts/cues** – posters were displayed in the cafeteria, at after-school programs, and in the community for visual reinforcement  **8.1 Behavioral practice/rehearsal** – taste tests  **8.2 Behavior substitution** – brainstorm about healthy snack/breakfast options that include FVs – brainstorm ways to be more physically active, or reduce television viewing time  **12.5 Adding objects to the environment** – FV serving size information poster, stickers  **Community participation/involvement** – families were given materials with guidelines, recipes, and tips  **Knowledge transfer** – increase knowledge in several areas, classroom-based nutrition and PA education lessons  **Active learning** – children conduct their own research; combine FV with mathematics; students write a persuasive letter to friends; students do presentations; students write a letter advocating for a change that they would like to see at their school or in their community; students use advertising techniques to develop a jingle or slogan; students identify the effect of food advertising on food preferences | **PA**: Survey consisting of the Self- Administered Physical Activity Check- list. Children reported how many minutes they spent doing each activity yesterday: before school, during school (classroom, physical education, and recess/lunch), and after school.  **DB**: 24-hour food diary, 24-hour recall interview | **PA**: ↔  At T1 compared to T0 the intervention group showed a significant increase in PA minutes during recess/lunch (5.1 minutes more) compared to the control group.  **DB**: ?  At T1 (post-intervention) compared to T0 the intervention group increased FV intake, while the FV intake of the control group decreased (mean difference in change 0.26 cups). Most of this difference came from the intervention group eating more whole fruits (0.18 cups more) than the control group from T0 to T1; a smaller amount was observed for vegetable intake (0.10 cups more). |
| Lent et al., 2014[45]; weak quality rating | This study aimed to impact healthy purchases in corner stores close to the school. Corner stores: (1) display study marketing materials; (2) stock a minimum of products targeted by the intervention (3) group healthier items for easy identification. The three main intervention components are: 1) seven classroom-based nutrition education lessons 2) a branded social marketing campaign 3) corner store-level initiatives including storeowner trainings, adding healthier items, and signage identifying healthy items  **1.3 Goal setting (outcome)** – goal setting taught by project staff  **12.1 Restructuring the physical environment** – change in prices - owners were encouraged to group healthy snacks together  **12.5 Adding objects to the environment** – 11 branded refrigerated barrels and one branded countertop refrigerator were provided to intervention stores – adding healthier items in stores, signage identifying healthy items – a branded social marketing campaign communicated messaging regarding healthy eating and well-being – logo was imprinted on small giveaways and banners and was displayed in corner stores – website, comic book and video  **Community participation/involvement** – youth leaders provided feedback on which healthy items to introduce and on marketing messaging – for store owners: training  **Knowledge transfer** – classroom-based nutrition education lessons on identifying healthy snacks | **DB**: corner store intercepts. Include: intercept interview (1-2 minutes), based on product’s category, size, name, quantity was determined the energy content, fat, sodium, carbohydrate, sugar, protein, fiber. | **DB**: ↔  No significant effects. |
| Mendoza et al. 2017[36]; weak quality rating | Bicycle trains consist of a group of children who cycle to school under supervision of an adult. At 'train stops' in the neighborhood children are being picked up and dropped off at set times.  **3.2 Social support** – study staff rode along the route  **4.1 Instruction on how to perform a behavior**, and  **6.1 Demonstration of the behavior**, and **8.1 Behavioral practice/rehearsal** – 2-3-hour professional bicycle riding safety course  **8.1 Behavioral practice/rehearsal** – several children learned to ride a bicycle for the very first time  **8.2 Behavior substitution**, and **8.4 Habit reversal** – the bicycle train was designed to instill an active lifestyle in children by promoting cycling to and from school over passive commuting  **8.3 Habit formation** – going by bicycle to school 5 days/week  **10.1 Material incentive (behavior)** – children were eligible to keep the study bicycles and equipment after study completion  **12.2 Restructuring the social environment** – bicycle train route based on children’s addresses with stops located along the route to school  **12.5 Adding objects to the environment** – participants were provided and fitted with (free) bicycles and safety equipment  **Knowledge transfer** – 2-3-hour professional bicycle riding safety course | **PA:** A questionnaire administered in one whole week the percentage of trips made to school by cycling. MVPA (minutes/day) measured by 1) a combination of accelerometers and GPS units (for cycling only) and 2) accelerometers only (for all PA activities except cycling). Devices were worn for 7 days. | **PA:** ↑  At T1 compared to T0 the intervention group had an absolute increase in mean percentage of daily commutes by cycling of 44.9% compared with controls. Intervention participants had an increase in mean MVPA of 21.6 minutes/day (95%CI=8.7, 34.6) compared to controls from T0 to T1. |
| Neumark-Sztainer et al., 2009[46]; weak quality rating | Theatre-based after-school program to reach ethnically diverse and low-income children and their parents with obesity prevention messages  **3.1 Social support (unspecified)** – the family component aimed to enhance home support for behavioral changes through positive reinforcement of healthy behaviors, parent-child participation in physical activities and availability of healthy foods – children were given an opportunity to share any behavioral changes they had made over the past week  **4.1 Instruction on how to perform a behavior** – parent postcard with information and interactive activities on a topic addressed in the after-school program  **6.1 Demonstration of the behavior**, and  **8.1 Behavioral practice/rehearsal** – participants furthered their physical activity skills by teaching their classmates and they learned exercises to do at home with their families  **5.2 Salience of consequences** – intervention messages were transformed into play scenes  **7.1 Prompts/cues** – weekly fun and fitness packs were sent home that included a healthy food with a simple recipe or fitness incentives for the family – a CD of the Ready. Set. ACTION! Songs was also sent home to foster dance at home – parent postcard  **13.4 Valued self-identity** – promoting a positive body image through interactive activities  **Community participation/involvement–** two family events – props sent home to parents  **Knowledge transfer** – the ACTivities focused on enhancing knowledge related to physical activity and healthy eating | **PA**: Self-report: hours/day after school and previous day  **DB**: 24-hr recall: energy intake (kcal/day), fruit/vegetable intake (number of servings/day), sweetened beverages (number of servings/day), **ST**: Self-report: TV viewing (hours/day) | **PA**: ↔  No significant effects  **DB**: ↔  No significant effects  **ST**: ↔  No significant effects |
| Nollen et al., 2014[34]; weak quality rating | This is a mobile technology intervention targeting nutrition and sedentary behavior of girls. Through their mobile phone girls had to set two daily goals and an accompanying plan for improving the behavior addressed in three four-week modules. The app gave them cues to actions and feedback to self-monitor goal attainment.  **1.1 Goal setting (behavior)**, and  **1.4 Action planning** – girls were required to set two daily goals and an accompanying plan for improving the behavior  **2.2 Feedback on behavior** – feedback and reinforcement on goal-attainment  **2.3 Self-monitoring of behavior** – cues to action and self-monitoring that prompted girls to self-monitor progress toward their goals  **7.1 Prompts/cues** – cues to action and self-monitoring that prompted girls to self-monitor progress toward their goals at five preselected times throughout the day  **10.3 Non-specific reward** – song-based reward system that provided girls one song per day if they responded to 80% of daily prompts | **DB**: 24-hour dietary recall multiple pass method to assess fruit/vegetables, sugar sweetened beverages  **ST**: Brief Questionnaire of Television Viewing and Computer Use | **DB**: ↔  No significant effects  **ST**: ↔  No significant effects |
| Salmon et al., 2008[57]; weak quality rating | The intervention is aimed at preventing excess weight gain, reduce likelihood of being overweight or obese, reduce screen time and promote PA. There are 3 intervention conditions: behavioral modification (BM), fundamental movement skills (FMS), or combination (BM/FMS). Intervention groups received 19 lessons, combination group double, delivered by a qualified PE teacher.  BM  **1.1 Goal setting (behavior)**, and  **1.4 Action planning** – the child selects the TV programs that he/she wants to watch and limits viewing to those programs  **1.8 Behavioral contract** – children signed a contract pledging to switch off one TV program per week over the next 4 weeks  **2.2 Feedback on behavior** – reinforce children’s awareness of their physical activity through the use of pedometers  **2.3 Self-monitoring of behavior** – children self-monitored the time they spent in sedentary behaviors and physical activity  **4.1 Instruction on how to perform a behavior**, and  **6.1 Demonstration of the behavior**, and  **8.1 Behavioral practice/rehearsal** – children participated in physical activities that they could easily perform at home on their own  **4.4 Behavioral experiments** – raise children’s awareness of the home and community environments through map drawing and photographic techniques  **5.1 Information about health consequences** – health benefits of physical activity were covered  **8.2 Behavior substitution** – children developed their own physical activities and games in which they could participate as an alternative to being sedentary  **8.6 Generalization of a target behavior** – children participated in physical activities that they could easily perform at home on their own  **8.7 Graded task** – children had to switch off one TV program per week  **9.2 Pros and cons** – children had to weigh up the positives and negatives of choosing between being active or sedentary  **10.2 Material reward (behavior)** – an intermittent reinforcement schedule was employed with children receiving a small reward for the return of each of the first four contracts and thereafter an intermittent reward for continuing to maintain the switch-off  **10.3 Non-specific reward –** children received a participation medal  **13.1 Identification of self as role model** – children presented their advocacy posters to children in the younger grades  **14.9 Reduce reward frequency** – intermittent reward for continuing to maintain the switch-off  **Community participation/involvement** – parents had to sign the contract each week  **Knowledge transfer** – lessons – raising awareness about advertisements on TV  **Active learning** – children write their own scripts, perform plays and design posters about choices to be active or sedentary based on real-life situations  FMS  **4.1 Instruction on how to perform the behavior**, and  **6.1 Demonstration of the behavior**, and  **8.1 Behavioral practice/rehearsal** – children were taught six skills: three object control skills and three locomotor skills | **PA**: Accelerometer: counts/day, time/day in MPA and VPA  **ST**: Questionnaire: TV viewing, computer use and playing electronic games (hours/minutes on weekdays and weekend days). | **PA**: BM vs control group ↔: counts/day β=47.0 (95%CI: 24.2, 69.8) at T1 (post-intervention) and β=7.5 (95%CI: 24.6, 70.4) at T2 (12-month follow up); VPA (min/day) β=2.8 (95%CI: 0.3, 5.4) at T1 and β=2.8 (95%CI: 0.2, 5.4) at T2. FMS vs control group ↑: counts/day β=76.7 (95%CI: 35.2, 118.0) at T1 β=76.1 (95%CI: 33.4, 118.9) at T2; MPA (min/day) β=10.4 (95%CI: 2.8, 18.1) at T1 and β=9.5 (95%CI: 1.4, 17.6) at T2; VPA(min/day) β=7.8 (95%CI: 3.4, 12.3) at T1 and β=7.7 (95%CI: 3.2, 12.1) at T2.  BM/FMS vs control group ↔: No significant effects  In boys: BM vs control group: counts/day β=61.6 (95%CI: 12.4, 110.9) at T2; VPA (min/day) β=4.5 (95%CI: 0.91, 8.0) at T1; VPA (min/day) β=4.4 (95%CI: 0.44, 8.4) at T2. FMS vs control group: counts per day β=112.8 (95%CI: 59.1, 166.6) at T1 and β=114.0 (95%CI: 52.8, 175.2) at T2; VPA (min/day) β=13.8 (95%CI: 8.7, 18.9) at T1 and β=13.8 (95%CI: 8.4, 19.1) at T2. BM/FMS vs control group: VPA β=5.7 (95%CI: 0.21, 11.2) at T1. In girls: BM vs control group: counts per day β=36.5 (95%CI: 0.29, 72.7) at T1 and β=29.3 (95%CI: 9.4, 69.1) at T2; MPA β=12.1 (95%CI: 3.9, 20.3) at T1 and β=11.1 (95%CI: 3.8, 18.4) at T2 FMS vs control group: No significant effects BM/FMS vs control group: No significant effects  **ST**: BM vs control group↔: TV viewing (min/week) β=229.3 (95%CI: 16.6, 442.0) at T1 and β=239.9 (95%CI: 27.6, 452.2) at T2;  FMS vs control group ↔: No significant effects BM/FMS vs control group ↔: No significant effects |
| Slusser et al., 2013[38]; weak quality rating | Staff training on nutrition education and PA; regular monitoring and technical assistance visits; after-school youth specialists were coached to increase students' opportunities to participate in MVPA; Catch Kids Club curriculum are 32 lessons on nutrition and skills to make healthy dietary and PA choices at school, in the community and at home  **3.1 Social support (unspecified)** – youth specialists were encouraged to serve as positive role models to the children  **4.1 Instruction on how to perform the behavior** – nutrition education – materials for parents  **4.1 Instruction on how to perform the behavior**, and  **6.1 Demonstration of the behavio**r, and  **8.1 Behavioral practice/rehearsal** – hands-on snack preparation activities  **12.2 Restructuring the social environment** – physical activity cards | **PA**: Catch Kids club questionnaire: physical activity behavior. Previous day physical activity recall (PDPAR): i.e. previous day recess and lunchtime activities, transport. Calculated into one score (including SB)  **DB**: Day in the Life Questionnaire: frequency of fruit, vegetable, snack food and juice intake (previous day) | **PA**: ↔ No significant effects  **DB**: ↔ At T1 (post-intervention) compared to T0 frequency of drinking juice lower in intervention group vs control group (0.18 vs 0.44); frequency of eating junk food lower in intervention group vs control group (-0.23 vs 0.01). |
| Springer et al., 2012[47]; weak quality rating | Students track the number of miles they walk or run along with the number of fruits and vegetables they eat. Successful completion of MK is 26.2 miles walking or running over 6 months and eating fruit or vegetables 5 times a day for 26 days for 1 month.  **1.1 Goal setting (behavior)**, and  **1.4 Action planning** – successful completion of MK is based on a set of targets  **2.3 Self-monitoring of behavior** – students track the number of miles they walk or run along with the number of fruits and vegetables they eat  **3.1 Social support (unspecified) –** promoting teacher and parent encouragement  **3.2 Social support (practical)** – teachers often assist students with the tracking of their miles and FVC  **8.1 Behavioral practice/rehearsal**, and  **8.3 Habit formation** – walking or running 26.2 miles over a 6-month period and eating fruit or vegetables 5 times a day for 26 days for 1 month  **10.10 Reward (outcome)** – students who complete the program receive a finisher t-shirt and those who attend the final mile run also receive a medal  **12.1 Restructuring the physical environment** – structured time is provided during recess, PE class, or other periods of the school day for students to walk or run  **12.5 Adding objects to the environment** – logo and bumper stickers, community signage such as advertising on buses, MK t-shirts, widely publicized book-end events  **Knowledge** **transfer** – presentations to school districts and PE teachers, information packets, promotion of school garden | **PA**: Active Kids Project questionnaire with items from PAC-Q, the Athletic Identity Questionnaire, the GEMS study: includes PA engagement. Items from the PAC-Q: 7-day recall items on running, walking and other leisure-time PA  **DB**: Active Kids Project questionnaire, includes items from SPAN survey and SIP 15 project: fruit and vegetable consumption (FVC) at home and at school. | **PA**: ↔  At T3 (post-intervention) compared to T0 (repeated measures) a higher mean number of times running in the intervention group vs the control group (4.38 and 3.83 respectively; standardized effect size 0.16). No effects on walking, recreational activity or global PA.  **DB**: ↔  At T3 compared to T0 a higher mean times of fruit intake in the intervention group vs control group (3.25 vs 2.96; standardized effect size 0.14) and vegetable (2.67 vs. 2.38; standardized effect size 0.14) intake per day, and higher vegetable intake during school lunch (2.66 vs 2.44). No differences for fruit juice consumption or frequency of eating fruit at school. |
| Trude et al., 2018[50]; weak quality rating | BHCK is a multilevel, multicomponent childhood obesity prevention trial that sought to modify the food environment on the policy, environmental, interpersonal and intrapersonal level. BHCK involves wholesalers, small food stores, recreation centers/peer-mentors, and social media. The intervention consists of three phases: 1) healthier beverages – promoting healthier alternatives to sugar sweetened beverages; 2) healthier snacks – promoting low-fat and low-sugar alternatives to unhealthy snacks; 3) healthier cooking – promoting cooking ingredients.  **1.1 Goal setting (behavior)** – goal setting for healthier food intake  **3.1 Social support (unspecified)** – interaction between parents is encouraged  **4.1 Instruction on how to perform the behavior** – storeowner training which aim to improve their knowledge of healthier food options and self-efficacy to be able to stock, prepare and sell their foods; taste tests and blind tasting challenges in stores  **4.1 Instruction on how to perform the behavior**, and  **6.1 Demonstration of the behavior**, and  **8.1 Behavioral practice/rehearsal** – cooking classes  **6.2 Social comparison** – participants are asked to share whether they were able to achieve weekly goals and advise other parents  **7.1 Prompts/cues** – caregivers receive a text message to encourage completion of an attainable and specific goal, subsequent text-messages offer support to help parents reach the goal, social media mirror the content of the text-messages  **9.1 Credible source –** youth-leaders are trained to deliver components of the intervention  **10.4 Social reward** – stores will be deemed a bronze, silver, gold or platinum certified healthy store if they successfully stock new, healthy food options and allow the BHCK team to promote the foods with their stores throughout the phases  **12.1 Restructuring the physical environment –** increase access to healthier foods by small retail and prepared food sources in the city, by increasing the stocking and sales of affordable healthy food options at local wholesalers that supply corner stores and carryout; wholesalers provide discounts on BHCK promoted food items to participating small food sources; at carryout storeowners are asked to make the default beverage option a healthier option, provide healthier side dishes, engage in healthier cooking methods and create a healthy combo meal on their menu  **12.5 Adding objects to the environment –** storeowners receive gift cards from small food sources; corner storeowners are asked to stock at least four new healthy food options; signage such as menu boards, shelf talkers, labels and posters  **Active learning** – the instructional period is followed by interactive games, activities, taste tests, and cooking classes to reinforce promotional messages; youth-leaders will deliver interactive sessions in the corner stores  **Community involvement/participation** – collaboration with key stakeholders, wholesalers, recreation centers, peer leaders, corner stores/carryout, families; the youth-leader intervention was developed in a participatory process with community partners and young people; youth leaders deliver intervention components in stores, via promotional materials, and on social media  **Knowledge transfer** – storeowner trainings to improve their knowledge of healthier food options and self-efficacy to be able to stock, prepare and sell their foods. | **DB:** Block Kids 2004 Food Frequency Questionnaire (consumption of 77 common food items in past 7 days) | **DB:** ↔ No significant effects |
| Vander Ploeg et al., 2014[53]; weak quality rating | Comprehensive school health approach, with actions in 4 pillars: social and physical environment, teaching and learning, healthy school policy, partnerships and services. Involves the placement of a full-time facilitator, to create and sustain supportive physical and social environments that cultivate a healthy lifestyle. Activities were organized, PA equipment in classrooms, improve access to after-school PA facilities and programs, support active transportation  **3.1 Social support (unspecified)** – student/parent activities  **4.1 Instruction on how to perform the behavior**, and  **6.1 Demonstration of the behavior**, and  **8.1 Behavioral practice/rehearsal** – a variety of enjoyable activity choices were offered weekly  **8.2 Behavior substitution** – improve traffic safety to promote and support active transportation  **12.1 Restructuring the physical environment** – improve access to after-school physical activity facilities and programs  **12.2 Restructuring the social environment** – placement of a full-time School Health Facilitator in each school  **12.5 Adding objects to the environment** – schools had easy and ready-to-use equipment in classrooms to facilitate increased activity  **Community participation/involvement** – student/parent activities – monthly school newsletters  **Knowledge transfer** – monthly school newsletters describing affordable, easily accessible, and seasonally appropriate activities for children to participate in outside of school | **PA**: Pedometer: daily physical activity level, i.e. steps. | **PA**: ↑  At T1 (post-intervention) compared to T0 steps/day increased with 13.0% more in the intervention group vs the control group, β=1399 (95%CI: 485, 2312). |
| Wang et al., 2019[37]; weak quality rating | Community-based behavioral intervention, delivered through Boys and Girls Clubs of America (BGCs). The intervention consists of 1) 12 group-based weekly sessions (1-h session, twice a week); 2) BGC open house event for youth and parents. Intervention activities consisted of three components: 1) health sessions for youth (knowledge, attitude, skills), 2) narrative sessions for youth (behavioral messages on SSB and water intake), 3) youth-led activities empowering youth as change agents (e.g. teaching parents knowledge and skills, sharing of narrative materials produced)  **1.1 Goal setting (behavior)** – through health and narrative sessions, e.g. set individual water intake goals  **1.2 Problem solving** – through health and narrative sessions, e.g. identify triggers for SSBs, brainstorm ways to avoid SSB’s, create video narratives to overcome barriers to drinking water  **2.3 Self-monitoring of behavior** – the booklet that child participants received included intervention activity worksheets, and water and SSB consumption tracking sheets  **4.1 Instruction on how to perform the behavior** – through health sessions, e.g. instruct youth on nutrition labels  **4.1 Instruction on how to perform the behavior**, and  **6.1 Demonstration of the behavior**, and  **8.1 Behavioral practice/rehearsal** – weekly health module and narrative module, where the narrative modules include intervention objectives and activities that reinforce the knowledge, attitudes, skills and behaviors targeted in the health component  **5.1 Information on health consequences** – health and narrative sessions, e.g. discuss benefits of drinking water  **8.2 Behavior substitution** – help youth identify non-sweetened alternative beverages to SSBs; encourage youth to try different beverage options  **8.6 Generalization of a target behavior** – health and narrative sessions  **10.2 Material reward** – celebrate youth program completion through distribution of certificates and prizes  **12.5 Adding objects to the environment** – child participants receive a reusable water bottle and a pictorial intervention booklet  **Active learning** – weekly health module and narrative module, where the narrative modules include intervention objectives and activities that reinforce the knowledge, attitudes, skills and behaviors targeted in the health component  **Community involvement/participation** – the booklet that child participants received included parent-child take-home activities | **DB:** Items adapted from the Youth Risk Behavior Surveillance (YRBS) survey and Youth Adolescent Food Frequency Questionnaire (consumption of SSBs, water, fast food, FV, in past 7 days)  **PA: I**tems of the YRBS survey (number of days engaged in at least 60min of MVPA per day over the past 7 days)  **ST:** Items of the YRBS survey (number of hours of screen time on an average school day) | **DB:** ↔  At T1 (2-month follow up) compared to T0 SSB intake decreased (servings/day) β=-0.97 (95%CI: -1.75, -0.20) and water intake (cups/day) increased β=1.23 (95%CI: 0.42, 2.05), in intervention group vs control group. At T2 (6-month follow-up) compared to T0 SSB intake decreased β=  -1.64 (95%CI: -2.52,  -0.76) and water intake increased β=1.31 (95%CI: 0.38, 2.23), in the intervention group vs the control group.  **PA:** ↔  No significant effects  **ST:** ↔ No significant effects |
| Wells et al., 2014[48]; weak quality rating | School garden program to increase physical activity and decrease sedentary behavior. Four components in the program: 1) the garden 2) access to a curriculum of 20 lessons 3) resources for the school 4) a garden implementation guide  **4.1 Instruction on how to perform the behavior**, and  **6.1 Demonstration of the behavior**, and  **8.1 Behavioral practice/rehearsal** – educators led activities in the garden such as planting, weeding and harvesting  **12.5 Adding objects to the environment** – the school garden  **Knowledge transfer** – information and implementation guide to the school – 20-lesson curriculum for children | **PA**: GEMS Activity Questionnaire (GAQ): information on physical activity (usually; yesterday) in 28 sports and physical activities (e.g. Volleyball, hiking, gymnastics, bicycling).  Accelerometer: physical activity (light PA, MPA, VPA, MVPA).  **SB**: GEMS Activity Questionnaire (GAQ): information on sedentary activities (usually; yesterday) in 7 sedentary activities (e.g. TV viewing, video gaming, computer games, board games, music listening). Accelerometer: physical activity (sedentary). | **PA**: ↔  At T1 (post-intervention) compared to T0 greater increase in % of time spent in MPA and MVPA in the intervention group vs the control group based on accelerometer data, mean diff=0.58 and 1.0, respectively.  **SB**: ↔  The intervention group showed less sedentary behavior (usually, not yesterday): mean diff=-0.19. No significant effects on accelerometer based SB. |

↔ indicates intervention overall not effective on at least 75% of the outcomes within the behavior, ↑ indicates intervention overall effective on at least 75% of the outcomes within the behavior, ?= unclear on the amount of outcome measures in the questionnaire
BM=behavioral modification, CG=control group, DB=dietary behavior, FMS=fundamental movement skills, FV=fruits and vegetables, IG=intervention group, MPA=moderate physical activity, MVPA=moderate to vigorous physical activity, PA=physical activity, SB=sedentary behavior, ST=screen time, T0=baseline, VPA=vigorous physical activity, vs=versus
*ST is reported when a study specifically focused on ST instead of on SB in general
